# Exploring grassroots feedback about cancer challenges in South Africa: a discussion of themes derived from content thematic analysis of 316 photo-narratives

**DOI:** 10.11604/pamj.2017.28.173.11894

**Published:** 2017-10-25

**Authors:** Lynn Barbara Edwards, Linda Estelle Greeff

**Affiliations:** 1 Psychologist in Private Practice, Cape Town, South Africa; 2 Clinical Oncology Social Worker, GVI Head Oncology Social Work, Cape Town, South Africa

**Keywords:** South Africa, cancer challenges, patient-centred care, advocacy, stigma

## Abstract

**Introduction:**

Cancer is an important health problem in Africa with projections that incidence could double by 2030. While sparse, the literature on cancer control in African low- and middle-income countries suggests poor cancer planning, overburdened services and poor outcomes. South Africa has established oncology health care services but also has low cancer awareness, poor cancer surveillance and widespread service challenges.

**Methods:**

Data for this study was derived from 316 photovoice interviews with cancer patients, families of cancer patients and oncology workers across South Africa. The objectives of the study were to collect first-hand feedback about cancer challenges and to develop recommendations for the improvement of cancer control strategies.

**Results:**

9 themes of cancer challenges were distinguished via thematic content analysis of the photo-narratives. The identified themes of cancer challenges were physical and treatment challenges, emotional, poor services, transport, finances, information, powerlessness, stigma, and schooling challenges.

**Conclusion:**

The findings of this study offer the patient and family perspective of cancer challenges as a valid contribution to our body of cancer knowledge. The 9 themes of cancer challenges profile the emotional, physical and social impact of cancer on patients and families, and offer detailed subjective information about problem occurrence in the trajectory of care. Recommendations following from the 9 themes of cancer challenges include training for improved patient-centred care standards, the need for cancer surveillance, innovative and locally appropriate cancer awareness campaigns, private and government health care partnerships and the development of psychosocial services. The advocating of findings and recommendations to influence cancer control strategies in South Africa, is indicated.

## Introduction

The relatively sparse literature on the status of cancer control in low- and middle-income African countries paints a picture of inadequate cancer planning and control, low public awareness and comorbidity with cancer related infectious diseases [1,2]. Resource constrained health services, widespread poverty, malnutrition, stigma and superstition are known to lead to frequent presentation of late stage disease with poor treatment outcomes and high mortality [3]. The adoption of Western habits and survival to older ages, is expected to increase the incidence of cancer in Africa [2,4] thereby placing a serious burden on cancer planning, control and services. South Africa (SA) has an ethnically diverse population of nearly 53 million people, 11 official languages [5], an established public and private health infrastructure and a low cancer surveillance level of 1% [2]. A host of socio economic problems, low cancer awareness, health systems shaped by complex political history [6] and competing health priorities, all contribute to widespread challenges for many cancer patients in their efforts to access cancer diagnostic, treatment and care services [7-9]. The objective of this study was to collect information about cancer-related challenges that were experienced or noted by patients, family members and health care workers in SA. Photovoice [10] was chosen as an interview methodology because it has been well-used in a variety of international settings for the gathering of public health information and cancer advocacy [11]. The advantage of using photovoice as an interview method for this study is that the narrative component could be used as data for the thematic analysis of cancer challenges, and the photovoice material could serve as evidence-based advocacy material. The purpose of the thematic analysis was to identify and describe grassroots cancer-related challenges and thereby contribute to what is known about the experience of coping with cancer in the SA context.

## Methods

From 2014 to 2016, 286 cancer patients and family members (called patient participants) and 30 health care participants were selected using convenience sampling from tertiary oncology units and cancer interim homes across SA. Ethical review and clearance was granted by the Ethics and Research Committee of the Medical Research Council of South Africa (EC006-3/2014) and strict guidelines for informed written consent were adhered to. All participants were interviewed via face-to-face interviews using photovoice methodology [10] and a semi-structured interview to gather information about age, gender, home town, treatment town, private or public medical service, cancer type and participant type (cancer patient, relative or oncology worker). Through the use of translators and trained interviewers, indigenous languages and illiteracy were accommodated, and informed consent explained. Responses to 2 open-ended research questions (i.e. 1. Did you experience cancer challenges, and if so what were they? 2. Are there any specific cancer challenges that you think need urgent advocacy?) were documented as colloquial narratives (called photo-narratives). Cameras were provided by the interviewers and participants were assisted in the taking, titling and explaining of the meaning of their photograph. The title, reason for choosing the photograph and the actual photographic image were then combined with the documented photo-narrative to become advocacy material (Annex 1, Annex 2). Advocacy material was then available for use in projects such as breast cancer exhibitions, as cancer information material and as evidence in stakeholder feedback. The photo-narratives were then subjected to inductive thematic coding [12] using 3 coders to promote convergence and inter-coder reliability [13]. The thematic analysis coded the raw photo-narratives into categories of similar reference and meaning, thereby creating 9 themes of cancer challenges and contributing to the objectives of the study.

## Results

### Participants

316 Participants were sampled and represented 105 towns across SA. 232 Patient participants used the public health system and 54 used private medical care. 138 Patient participants were male, 148 were female and 68 interviews referred to childhood cancer. Children with cancer were supported by parents in the interview process, or where children were too young, parents were interviewed on their behalf.

### Thematic content analysis findings

9 Themes of cancer challenges were identified i.e. emotional, physical and treatment, poor services, information, powerlessness, finances, transport, stigma and schooling challenges. 92% of patient participants described emotional challenges such as acute and ongoing anxiety, fear about the future and death, distress of a cancer diagnosis, physical suffering, loss experiences, emotional support problems and concerns about family. Some participants emphasised positive patient coping skills, and some health care participants highlighted the emotionally challenging nature of oncology work. 66% of participants reported physical and treatment challenges which included specific difficulties related to surgery, chemotherapy, radiotherapy, pain and other symptoms, quality of life issues, socioeconomic struggles and the crisis of cancer progression. 61% of participants reported poor service challenges such as low cancer knowledge at primary care clinics and regional hospitals, broken equipment, testing delays, referral backlogs, dirty facilities, and a lack of psychosocial and financial support services. A number of participants also mentioned gratitude and appreciation for positive patient-centred care. 47% of patient/family participants lived within 50 km of an oncology treatment centre and 22% of this cohort reported transport challenges. 53% lived further than 50 km and up to 1045km from a treatment centre, with 47% of this cohort reporting transport challenges. Types of transport challenges did not differ between the cohorts but were exacerbated by distance travelled. Transport challenges included the discomfort of long journeys, long waits for transport, logistical problems, separation from family and transport costs. The finance challenges theme (40%) highlighted vulnerable households where primary breadwinners had low incomes, small state grants or were unemployed. Financial stress, loss of income, loss of employment, the burden of additional costs, difficulties accessing financial support and medical aid challenges were reported as common and devastating for many. 19% of participants emphasised information challenges which related to insufficient understanding of the early signs of cancer, little information about treatment and support services, language barriers and access to information barriers. Receiving good information about cancer was noted as reassuring and empowering while 14% of participants indicated feelings of disempowerment due to inadequate discussion with clinicians, poor information about treatment options, lack of patient-centred care and unequal power relations in the medical setting.

Problems of cancer stigma were reported by 31% of participants. Beliefs that provoked stigma included the idea that cancer was contagious, caused by evil spirits, a death sentence and tainted those it affected. It was reported that cancer stigma led to families being reluctant to speak about cancer and contributed to patient-experiences of isolation, shame, fear, discrimination and rejection. The schooling challenges theme was commented on by 10% of participants and highlighted stigma as a concern in the school environment for children who had cancer. Many children with cancer reported that they missed school life, and most parents were concerned about disruptions to schooling. Schooling concerns were aggravated by lack of hospital school services, shortages of hospital teachers, language barriers and children feeling too ill to learn. In this study 6 children with cancer had dropped out of school, 3 had fallen behind and anticipated dropping out, 2 young parents dropped out of their own schooling and 2 dropped out of college to look after their children. Hometown teachers were a valued support when they stayed in contact with learners, and children benefitted from parents being able to assist with tutoring and homework.

## Discussion

The principle findings of this study are the identification of the 9 themes of cancer-related challenges as contributed by those who have either experienced cancer, have a family member with cancer, or work in cancer care. Information about problems as well as factors contributing to positive patient-centred care, were noted. Patient-centred care is characterised by responsiveness and respect for patient needs, and where patient values guide the clinical process [14].

African research of non-biomedical aspects of cancer is rare [2,11] and a strength of this study is that it includes service-user information in an area of public health where constrained services can benefit from practical feedback [3]. Further strengths of this study is that it offers the personal perspective of first-hand experience as a valid contribution to knowledge, and that the use of photovoice material enables strong, evidence-based support for the advocating of findings to stakeholders. Shortcomings of this study include that it is a relatively small sample of a national population, that each photovoice contribution represents only a chosen narrative of the participant's broader experience, and that the subjective nature of photovoice methodology and convenience sampling, constrain the generalisability of findings [15].

The limited documented research on challenges to help-seeking behaviour for cancer symptoms in SA has focused on small samples of hospital patients with uniform cancer types [16-18]. This study extends the parameters of previous research through a widely distributed national sample and the inclusion of contributions from patients with any cancer type, family members and oncology workers. Amongst other findings, this study confirms previous conclusions, that stigma, information, finances, transport, poor cancer knowledge at the primary care clinics can seriously impede help-seeking behaviour [15-18].

### Discussion of themes of cancer challenges

**Emotional challenges:** 92% Of patient participants indicated emotional challenges with shock, fear, anxiety, depression, adjustment and post-traumatic distress frequently reaching clinical levels [9, 19-21]. Physical and treatment hardships as well as socioeconomic issues such as lack of support, stigma, transport and financial struggles are documented causes of emotional distress [22, 23], and in line with other research [24,25], those with advanced cancer reported increased levels of trauma and isolation. Separation of families was an intense stressor for many, and this was particularly traumatic when parents and guardians were denied the right to stay with children who were admitted to hospital [26,27]. Generally, parents of children with cancer reported more emotional trauma than their children who were noted as coping well as long as they had the constant presence of a parent or guardian. Despite the high prevalence of emotional distress, absence of distress screening and minimal psychosocial services in this sample, many participants reported emotional resilience and highlighted the importance of good care-provider relationships in coping with cancer [28].

**Treatment and physical challenges**: Physical and treatment challenges refer to the complex psychosocial, physical and treatment experience of cancer. Reports of diagnostic and treatment delays, emotional and physical suffering, disruption to family life, difficulty maintaining employment, logistics of care, information and travel needs, all illustrate the complex nature of physical and treatment challenges. Lack of community nursing and palliative care services is a concern given the known frequency of pain as a cancer symptom [7], and the importance of access to pain relief with late stage cancer [29,30]. However, even while vulnerable to a high symptom burden [4, 29, 31] adult participants with advanced cancer emphasised emotional challenges such as the trauma of recurrence, isolation, shame and fear for the future, over physical challenges. This study confirms that cancer and cancer treatments can cause physical and emotional suffering such as general poor health, nausea, weakness, fatigue, difficulty with eating and psychological changes. It also highlights that permanent disfigurement, loss of speech, sight or hearing, amputations, catheters and colostomies can pose a serious threat to quality of life [4, 30-35]. A consequence of surgical treatment can be emotional pain and loss e.g. the loss of sexual function and intimacy following mastectomy or prostatectomy [35], the loss of voice after a laryngectomy [33], and the loss of sexual confidence following colostomy [32]. With “hard to hide” surgical interventions and impairment of essential life tasks such as eating and speaking, those with cancer of the head, neck and oral area, are particularly vulnerable to the trauma of loss [4, 34]. Challenges of chemotherapy were concerned with side effects, the need for chemotherapy to be available closer to home for rural cancer patients, and parental distress when children were having chemotherapy. Poor understanding of radiation therapy was reported as problematic, but radiotherapy machines repeatedly breaking down was particularly frightening for patients who feared that treatment delays could lead to poorer outcomes.

**Poor services challenges**: As evidenced by this and other studies [27, 36, 37], lack of patient-centred care is inextricably linked with poor services and constitutes a risk to outcomes. In addition, health care participants in this study spoke out about inadequate oncology training standards, the lack of continuous professional education and low cancer detection skills becoming standards of health service. Poor services in the form of low suspicion of cancer at the primary health care level and inefficient cancer referral pathways, are well documented barriers to cancer care in Africa [3, 16, 38], and feedback from this study supports previous findings that the trajectory of care in SA is saturated with service barriers and backlogs [3, 8, 37, 39]. This study confirms that when waiting periods are long, treatment inadequate or staff unpleasant, then cancer patients will frequently seek alternative care, even at great personal inconvenience or financial cost to themselves. In line with previous findings [3, 39-41], participants highlighted a broad range of factors related to poor services i.e. inadequate national health funding, low prioritising of cancer, inadequate national surveillance, problems with transport, lack of co-ordination of services, administrative inefficiencies, understaffing, low clinical suspicion of cancer, incorrect diagnosis, poor medical decision-making, low dissemination of information and low standards of patient-centred care. Poor services were exacerbated and aggravated by low patient resources and poverty [37,39], poor social acceptability of cancer, cultural beliefs, language barriers and illiteracy. Poor services are documented as constituting a risk to patient outcomes and also to adding a burden of waste and extra costs to health care systems [42]. While needing further research, it was noted from this study is that 8% of the adult cancer interviews and 68% of childhood cancer interviews reflected appreciation for good care, suggesting that childhood cancer participants fared better in the receipt of patient-centred care than adult patients. Interim home care was identified as a valuable non-governmental service for both adult and childhood cancer patients, and was a critical service for many non-urban cancer patients.

**Transport challenges**: In this study 57% of the patient participants lived more than 50 km from an oncology treatment centre (rural cohort) and the remaining 43% lived within 50 km of treatment facilities (urban cohort). While 22% of the urban cohort and 47% of the rural cohort indicated similar transport challenges, problems were exacerbated for the rural cohort. SA is a large country sometimes requiring patients to travel many hundreds of kilometres to tertiary oncology treatment centres. Reports of travel hardships included logistical challenges, transport delays, travel costs, being ill while travelling and the need for information about blankets, food and water to cope with the journey. It was commonly found that patients had to sleep overnight in chairs or on the floor waiting for transport, and also that many young and older cancer patients had the hardship of needing to walk many kilometres to pick up points to access transport to hospital appointments. Transport was difficult and even embarrassing for those with certain cancers, e.g. anus cancer, head and neck lesions and colostomy bags, and one participant from the rural town of Calvinia suggested that the decision to die with dignity near to family was preferable to the harsh demands of travelling for cancer treatment. The need to travel away to distant treatment centres often resulted in worry about children at home, finances and many days of absence from work for treatment that took a few hours [2]. Public transport or finances for family visits was frequently not available, and many patients were separated from their families for extended periods of time and wished for treatment to be available closer to their homes.

**Financial challenges**: Illness can be a livelihood shock for many vulnerable households [17, 39], and failure to afford transport and medical costs can contribute to problems with access to care and ultimately lead to poorer outcomes [37]. Unemployment, and disruption to work creates a serious threat to financial sustainability, especially for low income households [15,18,39]. State cash grants and free services are often the only social protection measures for the poor [39]. This study confirms that the costs of cancer care was not well addressed in discussions between patients and doctors [43] and that financial assistance was difficult to access due to administrative problems, lack of information and payment delays. Many cancer patients in this study lived in extremely low income households which is a factor that has been reported to increase the likelihood of non-compliance or defaulting on treatment [8,15,38,44]. Clinical weakness and inefficiencies at the primary health level has been reported to generate a cost burden for the poor of between 30–50% of their monthly income highlighting how poor services can increase financial distress and undermine help-seeking efforts [40].

Even for those who had access to free public health care, often additional expenses were unavoidable and included transport, accommodation, toiletries, airtime, child care costs, stoma products, nutritional needs, consultation fees and other medical costs. Money shortages frequently resulted in prolonged and distressing separation of families. In one instance a mother put her 2 children in a shelter when she went for treatment and another mother could not miss work resulting in her 11year old daughter travelling unaccompanied 403 kilometres from Carnarvon to Kimberly for chemotherapy. Rural patients were particularly vulnerable to financial distress due to the additional need for accommodation, subsistence, communication and other co-incidental expenses [38]. 17,5% of South Africans have medical aid cover [45] for access to private health care which is affordable to about 20% of the population [46]. 54 Participants indicated accessing private health services and reported challenges that related to not having medical aid, exhaustion of medical aid benefits, financial distress and struggles for treatment authorisations [43]. It was noted in this study that some patients with low incomes but who had medical aid as an employment benefit, could not supplement 'shortfalls' in medical aid cover and engaged in dangerous cost defraying measures such as taking less medication and missing medical appointments and procedures [43].

**Information challenges**: This study confirms previous findings that a lack of public information can shape health-seeking behaviour by contributing to low awareness of the warning signs of cancer and a reluctance to respond to cancer symptoms [18,36]. Low cancer knowledge by health workers at primary and secondary health facilities contribute to low cancer suspicion, delays in diagnosis and inefficient referral pathways to tertiary treatment [18,36]. As previously reported [40], the draconian practice [26] of excluding parents and guardians from children's examinations or from staying with their children in hospital, was noted as effectively denying parental access to information and raised questions about the rigor with which informed parental consent was obtained for children's medical procedures. While rarely occurring in tertiary treatment units, this practice was not uncommon in regional hospitals and illustrates the difficulty of patients or family members being sufficiently empowered to oppose institutional authority, even when policy is at odds with the spirit of human rights legislation [47,48]. As with previous findings [18,40], frequent failure by health care staff to give good information about cancer and treatments led to patient frustration, confusion and low confidence in the care system. Language barriers, absence of translation services and a lack of information material was compounded by multilingualism and illiteracy, and while some participants mentioned that they received good cancer information, the provision of patient information was not a consistent aspect of patient care.

**Powerlessness challenges**: Patient powerlessness can disable personal agency and undermine help- or health-seeking behaviour [18]. Factors contributing to disempowerment in this study included insufficient provision of information, lack of patient-centred care, stigma, poverty, lack of support and frustrations with referral to treatment pathways. Powerlessness was also related to medical staff being unavailable or unwilling to communicate, and health workers being perceived as uncaring and not listening. In this and other studies [40] it was found that positive interactions with staff such as good communication, respect, kindness and helpful suggestions were empowering and that withholding information, petty rules, disregarding and humiliating patient-experiences were disempowering and led to a breakdown in co-operation and trust between care-providers and patients. The expert position of health workers and the functional authority required to manage patients in a health care environment is understood to create an imbalance of power relations between medical workers and patients [49]. In this study, patients reported being comfortable with authoritative professionals but experienced dominative power as intimidating and destructive. The benefits of adequate information and good quality patient-centred care was noted in both well-resourced and resource-constrained health settings where advantages were anxiety reduction and patient empowerment. Standing up for cancer care needs for oneself or for a family member, also contributed to feelings of personal agency and positive empowerment [28].

**Stigma challenges**: Cancer stigma is a negative and intimidating social force that typically originates from poor information about cancer, and where sound cancer education is the critical counter-strategy [50]. Misinformation noted in this study included that cancer was a death sentence, was contagious and was an illness that only affected Westerners. Similar to other findings [37], cancer stigma encouraged non-disclosure of symptoms, introduced obstacles to care and isolation from support. Traditional healers were noted as an important community health care resource and it is suggested in this and other studies [8], that collaboration of public health workers with local healers could advance public health care in traditional and rural communities. Notwithstanding the potential benefits of collaboration, where beliefs promote dangerous misconceptions, these should be addressed through sensitive and respectful education and training [8,50]. As reported in previous studies, cancer stigma was found to include beliefs that it is wrong to touch your breast [16] that breast self-examination may cause cancer [37], that vaginal bleeding is caused by witchcraft [36] and that cancer is a source of shame or a curse [18]. Taboos, social stigma and the threat of abandonment, has long been found to discourage help-seeking behaviour [44,51] and in this study 6 participants were abandoned by partners due to cancer.

**Schooling challenges**: Interruptions to normal school life, developmental milestones and separation from friends were problematic for many children with cancer [27]. The most educationally vulnerable children were those whose parents could not assist with homework, and where contact with hometown teachers was not maintained. School services were available in some of the larger hospitals but shortages of teachers, inconsistent student contact, language barriers etc., were challenges. 6 Children from this study had already dropped out of all schooling, and a further 3 were doubtful about returning to school. Cancer stigma at school was cited as a problem, and some parents kept their children home to protect them. Threats to adult schooling were also identified with 2 young parents abandoning their schooling and 2 abandoning tertiary education to be with their children. 4 Adult patient-participants reported disruption to their children's schooling due to demands of cancer care and 4 young adult patients dropped out of tertiary education due to the burden of cancer treatment.

**List of recommendations** (**Table 1**): Distress screening and psychosocial support services should be a standard of oncology care and subject to quality control; Standards of patient-centred care in oncology should be improved through training and professional development; Targeted training in cancer-mindedness and clinical skills at the primary and secondary levels of care is strongly indicated; Regionally centralised oncology diagnostic centres would support efficient screening and testing; Public-private partnerships to broaden service networks and make cancer care regionally accessible; Transport services need to be a more extensive and patient-centred service; Financial aid systems should be routinely and easily accessible; African-appropriate public cancer awareness programs need innovative design and implementation; Collaboration with traditional leaders and healers is an appropriate response to cultural patterns of health-seeking behaviour; Vibrant hospital schooling that collaborates with home-school teachers is indicated.

**Table 1:** 9 themes of cancer challenges: main findings & recommendations

Emotional challenges	Recommendations
Clinical levels of distress	Clinical level of distress should be diagnosed and referred
Absence of distress screening	Distress screening to be a standard of care
Lack of social support services	psychosocial support services should be mandatory in all cancer care units
**Physical and treatment challenges**	
Physical and treatment challenges can profoundly impact quality of life	Good patient centred care and clinical expertise to be upskilled
**Poor services challenges**	
Poor cancer knowledge at primary care clinics and regional hospitals contribute to delays in referrals and to poor outcomes	Training of primary and secondary care staff to be cancer-minded
Testing delays, referral backlogs impact poor outcomes	Strategically placed diagnostic centres with centralised testing equipment needed for the efficient and early detection of cancer
**Transport/travel challenges**	
Psychosocial impact of travelling away from home to tertiary oncology treatment units (rural patient highly impacted)	The need for public private partnerships to provide cancer services nearer to patients' homes, (especially for rural patients)
Logistical and quality of life travel hardships	Transport services need to be better co-ordinated and more patient-centred
**Finances challenges**	
Low income, unemployment and poverty widespread and impacts access to treatment	Service delivery needs to be decentralised with easy access to efficient services
Minimal access to financial support	Develop financial aid systems
**Information challenges**	
Public health issue where the general public has is insufficient understanding of early signs of cancer	Innovative public awareness programs (e.g. cancer education in schools, Pocket Cancer mobile phone project)
Many barriers to information	Need for medical translators and routine communication of patient information
**Powerlessness challenges**	
Lack of information is disempowering and impacts compliance and outcomes	Patient-centred care to be a performance standard with institutional quality control
**Stigma challenges**	
Lack of information or misinformation can provoke cancer stigma	Good public health cancer education targeting misinformation that causes stigma
Cultural factors may provoke cancer stigma	Collaboration and inclusion of traditional leaders and healers in public cancer care
**Schooling challenges**	
Hospital schooling not consistently available and not well resourced	Support for the establishment and maintenance of strong hospital schooling
Barriers to schooling e.g. stigma, finances, absence from school, parents unable to tutor, rural children having added disadvantage etc.	Childhood cancers requiring long term treatments need targeted psychosocial support services to protect the education and learning of young cancer patients

## Conclusion

The key findings of this study are the 9 themes of cancer challenges with major contributions being information about the severity of emotional stress, poor psychosocial services, lack of suspicion of cancer by health care workers and the urgent need for widespread implementation of patient-centred care standards. The ultimate purpose of this study is to use the findings to contribute to improvements in grassroots cancer care in SA and to offer feedback to a variety of stakeholders including the highest levels of government for improved national cancer planning and control.

### What is known about this topic

- SA has a developed, but resource-constrained, public health system where socioeconomic problems, low cancer awareness, wide population distribution and inefficient cancer detection services are widespread;
- Low cancer awareness and knowledge at primary health clinics is a serious obstacle to cancer care for the most under-resourced members of the population;
- Little information is available on the grassroots challenges of cancer in SA, and evidence-based information is needed to advocate for improved cancer planning and control.

### What this study adds

- Cancer patients and their families offer an important contribution to knowledge of the cancer experience in SA;
- Evidence-based information is provided in a rarely researched area of public health concern;
- A summary of recommendations, informed by the findings of this study, is provided to provoke practical solution-focussed thinking in cancer advocacy, planning and control.

## Competing interests

The authors declare no competing interest.

## Annexes

**Annex 1**: An example of photovoice interview material used in a cancer advocacy presentation to the National Health Department

**Annex 2**: An example of photovoice interview material used in a reference book for parents of children with cancer
